# First person – Tiantian Ji and Lina Zhang

**DOI:** 10.1242/dmm.041798

**Published:** 2019-08-29

## Abstract

First Person is a series of interviews with the first authors of a selection of papers published in Disease Models & Mechanisms (DMM), helping early-career researchers promote themselves alongside their papers. Tiantian Ji and Lina Zhang are co-first authors on ‘ Dynamic MAPK signaling activity underlies a transition from growth arrest to proliferation in *Drosophila scribble* mutant tumors’, published in DMM. Tiantian and Lina are both PhD students in the lab of Yan Yan at The Hong Kong University of Science and Technology Clear Water Bay, Hong Kong. Tiantian is investigating tumor heterogeneity and dynamic signaling activity during tumor progression, and Lina is investigating the mechanism of dynamic MAPK signaling during fly tumor development.


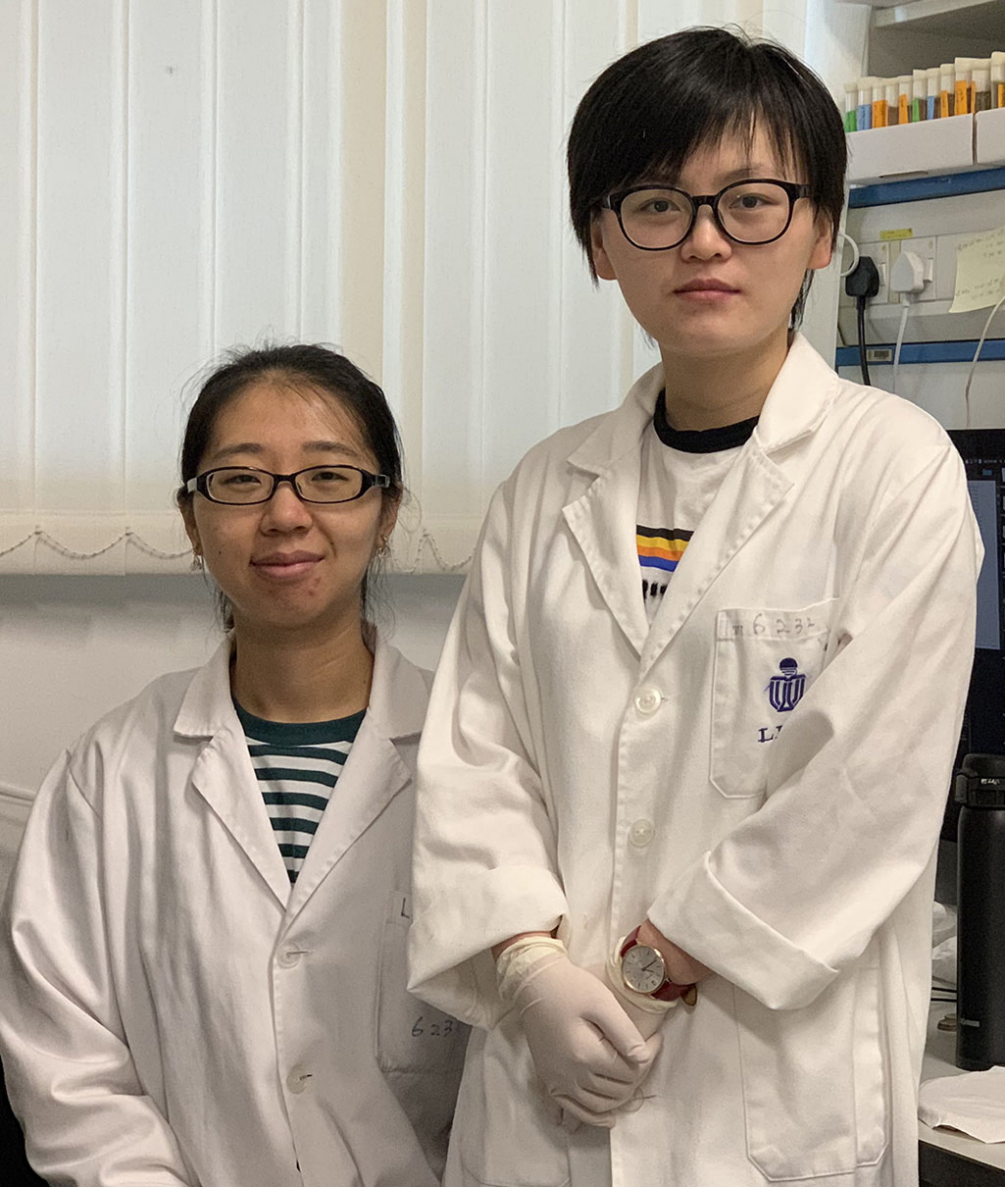


**Tiantian Ji and Lina Zhang**

**How would you explain the main findings of your paper to non-scientific family and friends?**

When you compare a patient tumor sample and normal tissue, you will notice that normal tissue architecture is often very messed up in the tumor sample. Scientists now understand that disruption of architecture is not just a passive consequence of tumor growth but has substantial and direct influences on tumor growth. Our study started with using a fruit fly tumor model to understand the tumorigenesis processes induced by loss of tissue architecture. To our surprise, we found that, in this simple fly tumor model, tumors evolve over time just like human tumors would do, therefore giving us an opportunity to study how tumors evolve in a simple model.

**What are the potential implications of these results for your field of research?**

There are two major implications from these results. First, our data showed that fly tumors are more plastic and complex on the temporal axis than we previously thought. Second, we found that many conserved signals regulating tumor growth are quantitative. We demonstrated that JNK and ERK signaling show quantitative changes over time and these changes directly influence tumor growth. Many other conserved signalling pathways, including JAK/STAT and Notch, also show similar temporal patterns in these tumor models, and it will be interesting to further dissect the functional consequences of quantitative changes of these signals on tumor progression.

“The fly tumor models are very similar to human epithelial tumors in many aspects.”

**What are the main advantages and drawbacks of the model system you have used as it relates to the disease you are investigating?**

Fly tumor model systems are really well-established by a dedicated community. The fly tumor models are very similar to human epithelial tumors in many aspects. And the system has a lot of tools available for sophisticated genetics, which is difficult to achieve in other systems. Also fly tumors are cheap to grow and they grow very fast, over 2 weeks instead of a few months or years, so that is advantageous for longitudinal studies. The major drawback is that the model system is perhaps less complex compared with human tumors. For example, fruit flies do not have a full adaptive immune system like humans do so it would not be suitable for modeling the interactions between tumors and the adaptive immune system.

**What has surprised you the most while conducting your research?**

The fly tumor model we used is a well-studied model, and we are most surprised that quantitative measurements are really powerful and led us to new findings.

“Traditional genetic approaches often simplify analysis of these signals using an on/off switch model.”

**Describe what you think is the most significant challenge impacting your research at this time and how will this be addressed over the next 10 years?**

Now we know that many conserved signaling pathways involved in tumorigenesis processes are quantitative and these quantitative features have a lot of influences on biological processes. Traditional genetic approaches often simplify analysis of these signals using an on/off switch model. One challenge is being able to develop tools to quantitatively control these signals in a well-defined and measurable manner and add quantitative features into our signal network models. The current development of single-cell techniques and optogenetic tools are very helpful for this goal.

**Figure:**
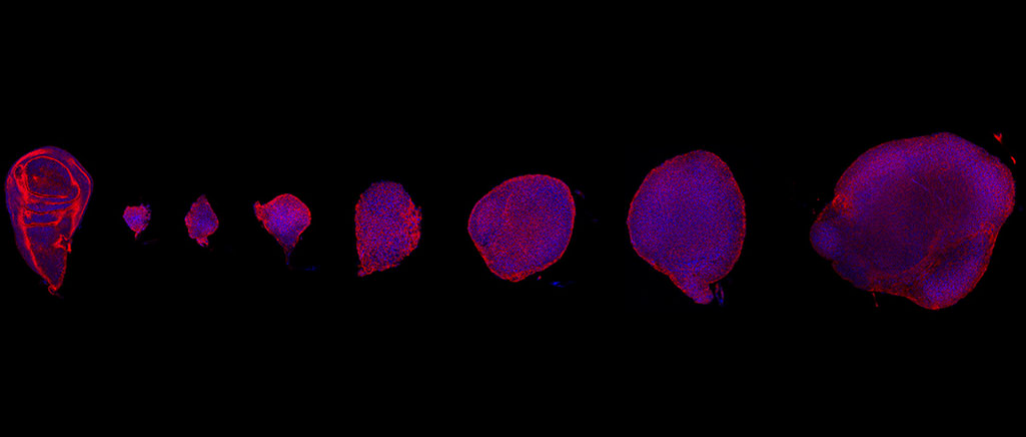
**An arrangement the normal and *scrib* mutant wing imaginal discs of different ages and sizes. The wing imaginal discs are stained with phalloidin and Hoechst to visualize F-actin and DNA.**

**What changes do you think could improve the professional lives of early-career scientists?**

TJ: I think it would be very helpful to have some funding opportunities open to graduate students.

LZ: I wish that there were more opportunities for exchange programs. I think that joining an exchange program is a good chance for PhD students to gain new expertise and ideas outside their own lab.

**What's next for you?**

TJ: I am quite interested in early-stage diagnosis of cancer and personalized medicine. After I graduate with my PhD degree, I think I would like to explore those areas both in academia and industry.

LZ: I am interested in tumor evolution and aging. I think that the current development of single-cell techniques and systems biology approaches is interesting. These are the factors I consider as I graduate with my PhD degree and look for a postdoc laboratory.
